# PP51 Critical Analysis Of Budget Impact Analyses For Health Technologies In The Brazilian Supplementary Health System

**DOI:** 10.1017/S0266462325102705

**Published:** 2025-12-29

**Authors:** Marina Meneses Aziz, Tayná Felicissimo Gomes de Souza Bandeira, Hérica Núbia Cardoso Cirilo, Maria Angélica Pires Ferreira

## Abstract

**Introduction:**

Budget impact analyses are essential for decision-making processes regarding the incorporation of technologies into healthcare systems. Despite advancements driven by the National Committee for Health Technology Incorporation (CONITEC), the Brazilian Network for Health Technology Assessment (REBRATS), and the National Supplementary Health Agency (ANS), challenges persist in evaluating the economic impact of new technologies, including methodological inconsistencies in submitted dossiers.

**Methods:**

Reports on the critical analysis of dossiers submitted to the ANS prepared by a health technology assessment center between December 2022 and December 2024 were analyzed. Criticisms of the submitted dossiers were categorized into eleven topics: (i) analytical model; (ii) reference scenario; (iii) alternative scenario; (iv) time horizon; (v) target population; (vi) direct costs; (vii) market behavior; (viii) sensitivity analysis; (ix) input data; (x) output data; and (xi) final decision. Descriptive and quantitative data were used to identify patterns and methodological gaps.

**Results:**

The critical analysis identified recurring issues, including underestimated target populations in 50 percent of cases and inadequate direct cost evaluations in 40 percent. These inadequacies were often linked to outdated data sources, such as the Painel dos Dados do TISS database. Analytical models were deemed adequate in 70 percent of cases, whereas sensitivity analyses were insufficient in 30 percent of cases. Market behavior projections were conservatively estimated in 60 percent of dossiers, affecting the accuracy of budget impact projections. These methodological inconsistencies hindered the reproducibility of analyses and compromised evidence-based decision-making.

**Conclusions:**

The findings highlighted the need for methodological standardization in dossiers submitted to the ANS, emphasizing the need to update data sources and ensure transparency in budget impact calculations. These improvements could enhance evaluation validity and support better decision-making for technology incorporation. Future research should address strategies to reduce uncertainties and inconsistencies in analytical models and cost estimations.

